# A systematic review on improving cognition in schizophrenia: which is the more commonly used type of training, practice or strategy learning?

**DOI:** 10.1186/1471-244X-14-139

**Published:** 2014-05-14

**Authors:** Karine Paquin, Alexa Larouche Wilson, Caroline Cellard, Tania Lecomte, Stéphane Potvin

**Affiliations:** 1 Psychology Department, University of Montreal, Montreal, Canada; 2 Department of Psychology, Concordia University, Montreal, Canada; 3 School of Psychology, Laval University, Quebec, Canada

**Keywords:** Schizophrenia, Explicit, Implicit, Training, Cognition, Sociocognition, Neurocognition

## Abstract

**Background:**

The purpose of this article was to conduct a review of the types of training offered to people with schizophrenia in order to help them develop strategies to cope with or compensate for neurocognitive or sociocognitive deficits.

**Methods:**

We conducted a search of the literature using keywords such as “schizophrenia”, “training”, and “cognition” with the most popular databases of peer-reviewed journals.

**Results:**

We reviewed 99 controlled studies in total (though nine did not have a control condition). We found that drill and practice training is used more often to retrain neurocognitive deficits while drill and strategy training is used more frequently in the context of sociocognitive remediation.

**Conclusions:**

Hypotheses are suggested to better understand those results and future research is recommended to compare drill and strategy with drill and practice training for both social and neurocognitive deficits in schizophrenia.

## Background

About 80% of individuals with a diagnosis of schizophrenia struggle with a variety of neurocognitive and sociocognitive deficits [1,2]. The neurocognitive domains typically affected include speed of processing, attention/vigilance, working memory, verbal learning, reasoning and problem solving [3,4], whereas social cue perception, affect recognition, attribution, and theory of mind are the sociocognitive domains most affected [5,6]. Cognitive dysfunctions are considered to be core features of schizophrenia, since they are strongly correlated with poor functional outcome [7-9] as well as being better predictors of general outcome and rehabilitation than positive symptoms [10,11]. Although pharmacological and psychological treatments can effectively reduce [12] positive symptoms of schizophrenia, they do little to improve cognition [7]. Thus, using cognitive retraining or remediation to create significant improvements has received more attention in recent years [7,13]. According to T Wykes, V Huddy, C Cellard, SR McGurk and P Czobor [14], there are two types of training: 1) “drill and practice,” where there is no explicit component, meaning that learning is based on repeating a task that becomes gradually more difficult and where participants implicitly learn the strategy by trial and error, and 2) “drill and strategy,” where the focus is to teach the explicit use of a determined strategy (see also [12]). While explicit learning impairments have been consistently reported in schizophrenia literature [15,16], there is still a debate over impairments to implicit learning. For example, some studies report that implicit learning is intact for tasks such as probabilistic classification learning (e.g., [17]), weather prediction (e.g., [18]), and artificial grammar learning (e.g., [19]), while others report an impairment in colour pattern learning but not in letter string learning [20]. Adding to this conundrum are a variety of different training procedures currently being tested, both for drill and strategy (includes explicit and implicit learning) and for drill and practice (implicit learning only). These training procedures focus on a variety of different targets therefore, in this review, we will focus on neurocognitive and sociocognitive domains. For this reason we will not include studies aiming solely to reduce positive or negative symptoms or to improve upon social skills. Contrary to the recently published meta-analyses focusing on efficacy of cognitive training [14,21], this review will analyze and describe which training paradigms were most used to improve neurocognitive and sociocognitive deficits, whether they be drill and practice or drill and strategy methods.

## Methods

### Review protocol

Inclusion criteria: 1) outcome: either neurocognition or sociocognition, 2) date and journal: peer-reviewed journals from 1995 up to 2013, 3) language: English or French, 4) diagnosis: majority (≥70%) of participants with a schizophrenia diagnosis (others include schizoaffective disorders and first-episode psychosis). We excluded all training types that aimed solely to reduce positive or negative symptoms, improve social skills, increase metacognition, etc. Nevertheless, studies that targeted sociocognition or neurocognition while also aiming to reduce symptoms or improve social skills as secondary objective, were included. Finally, we removed studies that used the training or remediation for evaluation rather than for treatment (i.e., studies assessing the deficits at baseline with no intention of remediation or intervention) as well as meta-analyses and reviews. Our goal was to review studies that had a therapeutic outcome. Since the main objective of our article is to provide a descriptive listing of the training offered and not to conduct an efficacy analysis, we included studies that did not have control conditions. Given the large number of articles included (n = 99), and the fact that our definitions of the types of training were inclusive, the first three authors read, classified, and compared their ratings for each article to ensure reliability of the results. Articles were classified in two categories, according to the targeted deficits: i) Sociocognitive, which included topics such as emotional recognition, Theory of Mind, attributional style, and social cue recognition; ii) Neurocognitive, which included areas such as executive functioning, memory and attention. Importantly, social functioning was excluded from the dichotomy of classification as most, if not all studies, ultimately aim to improve upon work and functional outcomes of individuals. Furthermore, we compared the results of our literature search with articles listed in the meta-analyses of T Wykes, V Huddy, C Cellard, SR McGurk and P Czobor [14], O Grynszpan, S Perbal, A Pelissolo, P Fossati, R Jouvent, S Dubal and F Perez-Diaz [22] and A Medalia and AM Saperstein [23] to ensure that we did not miss any relevant articles.

### Article retrieval

We conducted a literature review using the following databases: PsychINFO (1995 to May 2013), MEDLINE (R) (1995 to May 2013) and MEDLINE Daily Update (R). Using the title keywords “schizophrenia and (training or remediation or intervention or practice) and (soci*^a^ or neuro* or cogniti* or metacogniti* or problem-solving or visual or memory)” , we obtained 465 results from all databases. To ensure further precision we added the following filters: a) “limit to English and French language” (to ensure understanding of the content) which yielded 172 results, b) “limit to peer-reviewed journals” resulting in 164 results. The final manipulation was to remove all duplicates, which left us with a total of 121 articles to investigate. Upon final removal of all articles that did not meet our criteria, we reviewed 99 articles. The last date of search for articles was January 2014.

## Results

Results are presented in Tables 1, 2 and 3, divided according to the aim of the studies: improving neurocognitive deficits, sociocognitive deficits or both. These were further subdivided by either drill and practice or drill and strategy training methods. First, we will describe the studies that focus on a single area of cognition (i.e., Table 1 for neurocognition and Table 2 for sociocognition) as treatment targets and that used a single training type (drill and practice or drill and strategy). Then, we will describe the results of studies with multiple aims in terms of neurocognitive and sociocognitive deficits (Table 3). There is an important distinction to be made between the targeted deficits – which is how we classified the studies between neurocognition, sociocognition, or both – and the measured variables. Indeed, it is often the case that a variable is measured to assess the impact of the training without having been specifically targeted by the training, which, therefore, gives a sense of the generalization of the results. As seen more explicitly in Table 2, many of the studies aiming to improve sociocognition also measure the impact of the training on more neurocognitive variables.

**Table 1:** Training to improve neurocognitive deficits

**DRILL AND STRATEGY**					
**Authors**	**Targeted deficits**	**Type of training**	**Measured variables**	**Results**	**Control and samples**
[24]	Memory and problem solving	Cognitive Remediation (CR) and Treatment-As-Usual (TAU)	Psychiatric symptoms	Both CR groups improved on the Positive, negative and general psychopathology subscales but also on the Positive and Depression factors	Control group N = 54
[25]	Autobiographical memory	Group therapy and exercises to recollect specific events	Autobiographical memory, executive functioning	Improvements on the variables that were preserved after 3 months	Placebo group N = 27
[26]	Cognitive deficits, and transfert to functional competence	CR + skills training CR + TAU Skills training + TAU	Cognitive performance (reasoning, problem solving, processing speed, verbal memory, working memory) Social competence, functional competence, real-world functional behaviour	CR produced robust improvements in neurocognition, but not after functional skills training. Social competence improved with both trainings. Functional competence higher and more durable with combined treatment. Functional competence and real-world behavior was more likely when supplemental skills training and cognitive remediation were combined.	Control group N = 107
[27]	Neurocognition and transfert to social competence	CR and Functional Adaptation skills training (FAST) Control: FAST or CR	Functional competence, information processing, verbal fluency, working memory, executive functioning, verbal memory	The early-course group had larger improvements in measures of processing speed and executive functions, adaptive competence and real-world work skills. Verbal memory, verbal fluency and social competence did not improve	None N = 39
[28]	Neurocognition at large	CR and one-on-one training and guided practice	Attention, working and episodic memory, executive functioning, processing speed, everyday community functioning	No improvements were found	Placebo group N = 69
[29]	Psychiatric symptoms and cognition (episodic memory and attention)	Neurcognitive Enhancement Therapy (NET) + Work therapy and Verbal memory task based on a dichotic listening (DL) with distracter paradigm NET + Work therapy alone	Symptoms, attention and memory	Significant effect on memory but not on attention or symptoms. nor at 6 months follow up	Control group N = 125
[30]	Attention, memory and executive functioning	CR and group therapy	Verbal learning and memory, executive functioning, visual learning and memory, depression, positive and negative symptoms	Significant improvements in neuropsychological functioning, depression and negative symptoms of schizophrenia after CRT	Control group N = 42
[31]	Executive functioning	Cognitive Adaptation Training (CAT) applied to integrated treatment (IT) consisting of assertive community treatment (ACT)	Social functioning, symptoms and quality of life; executive functioning	Improved social functioning and compliance with IT and ACT. No solid evidence demonstrating that IT improves when adding CAT	Control group N = 62
[32]	Verbal and visual memory, sustained attention and executive functioning	CR with Neuropsychological Educational Approach to Remediation (NEAR)	Processing speed, executive functioning, sustained attention, verbal memory, visual memory, reasoning/cognitive flexibility, social/occupational functioning, life skills, quality of life, self-esteem	Experimental group showed improvement in all variables, gains maintained after 4 months	Control group N = 40
[33]	Verbal memory, working memory, motor speed, verbal fluency, attention, processing speed and executive functioning	CR with NEAR	Verbal memory,working memory, motor speed, verbal fluency, attention and speed of information processing, executive functioning	Improvement in all outcomes compared to control with CR	Control group N = 51
[34]	Cognitive deficits to improve work outcomes	Errorless learning Conventional instruction	Work performance, job tenure, personal well-being (self-esteem, job satisfaction, work stress)	The patients in the errorless learning group performed better on work performance	Control group N=40
[35]	Neurocognition at large	Cognitive (CR) and supported education	Self-esteem, short term memory, verbal learning and memory, executive functioning, sustained attention, psychomotor speed, educational attainment	CR can be successfully integrated into an educational setting. Improvements in concentration , learning, some aspects of executive functioning, psychosis symptomatology	None N=16
[36]	Cognitive deficits to improve work outcomes	Thinking Skills for Work Program (TSWP) + Supported Employment (SE) and Supported Employment only	Attention, psychomotor speed, information processing speed, verbal learning and memory, executive functioning, premorbid academic achievement, symptoms, employment outcomes	For TSWP+SE, improvement in executive functioning and in the composite cognition score. Improved significantly more on Depression and Autistic preoccupation (symptoms). Participants were significantly more likely to work, worked more hours and earned more wages	Control group N = 44
[37]	Cognitive deficits to improve work outcomes	Thinking Skills for Work Program (TSWP) + Supported Employment (SE) and Supported Employment only	Work outcomes	In TSWP+SE, over 2-3 years, participants were more likely to work, held more jobs, worked more weeks, worked more hours, and earned more wages. Cognitive functioning and symptoms not assessed.	Control group N = 44
[38]	Problem-solving	Computer-assisted problem-solving remediation (PS), memory remediation or TAU	Problem-solving, memory, verbal knowledge, independent living	PS improved problem solving skills	Control group N = 54
[39]	Cognitive differentiation, social perception, communication, social skills, and interpersonal problem solving	Integrated Psychological Therapy (IPT)	intellectual ability, memory, verbal fluency, executive functioning and psychosocial functioning	Improvement in memory and executive functioning for those with cognitive impairments	Control group N = 27
[40]	Social functioning and neurocognitive deficits	CR and Cognitive Behavior Therapy (CBT) for control	Working memory, psychomotor speed, verbal memory, nonverbal memory, and executive functioning, and social functioning	Overall improvement in neurcognition especially in verbal and nonverbal memory and executive functioning. Improvement in social functioning	Control group N = 40
[41]	Verbal and working memory, selective attention and semantic fluency	CR	Verbal and working memory, speed/coordination, selection attention, semantic and letter fluency, executive functioning, sustained attention, interpersonal relations, instrumental role, self-directedness	3, 6 and months follow up: improvements in attention, psychomotor coordination, cognitive flexibility	Placebo condition N = 100
[42]	Memory and executive functioning	One program including 1) paper-and-pencil training 2) computer exercises	Visual attention, cognitive flexibility, sustained attention, inhibition, working memory, long-term verbal memory, executive functioning, planning	CR showed improvements in neuro- and socio-cognitive functions but not on arousal or cognitive flexibility	Placebo group N = 59
[43]	Attention	Attention Process Training (APT) and attention-shaping procedure after	Verbal learning, sustained attention	Dramatic improvement in attentiveness in APT but attention-shaping procedure appears to account for the change	Control group N = 31
[44]	Neurocognition linked to social competence and behavior	Integrated Psychological Therapy (IPT), supportive therapy and TAU	Social competence, pre-attentional processing, attention, memory, executive functioning and symptoms	IPT improved social competence only	Control group N = 90
[45]	Memory, attention, vigilance, executive functioning	CR alone or CR+pharmacotherapy	Attention, learning, memory, executive functioning, functional capacity, negative symptoms, subjective quality of life	CR improved verbal and visual memory at 3 months, not maintained at 6 months. Verbal learning, executive functioning and attention improved at 6 months. Quality of life improvements at 3 months, increased at 6 months	Control group N = 38
[46]	Cognitive deficits and negative symptoms	Cognitive strategy training (CAST) and training of self-management skills for negative symptoms (TSSN)	Attention, verbal memory and planning, social withdrawal/social anhedonia, lack of drive, affect flattening	CAST=Greater improvement on attention and verbal memory but not planning ability. Higher job placement TSSN=no improvement in negative symptoms	Control group N = 138
[47]	Memory, cognitive flexibility and planning	Neurocognitive remediation and intensive occupational therapy (control)	Cognitive flexibility, planning and working memory. Social behaviour, self-esteem	Improvements in cognitive flexibility and working memory no changes in symptoms or social functioning, 6 month follow up	Control group N = 33
[48]	Memory, cognitive flexibility and planning	CR and Intensive occupational therapy	Memory, working memory, cognitive flexibility, response inhibition, planning, symptoms and functioning, self-esteem	Effects of CR at follow-up are still significant on working memory, there were no more effects on self-esteem, 3 and 6 month follow up	Control group N = 33
[49]	Memory, cognitive flexibility and planning	CR and TAU	Working Memory, cognitive flexibility, and planning, Secondary: self-esteem, positive and negative symptoms, social functioning	Improvement in working memory and cognitive flexibility, Memory improvement predicted improvement in social functioning.	Control Group N = 85
[50]	Memory, cognitive flexibility and planning	CR with remembering, complex planning, problem-solving and TAU	Memory, cognitive flexibility, planning, social behaviour, quality of life, self-esteem	CR improved cognitive flexibility, social functioning, 14 et 18 weeks follow up	Control group N = 40
**DRILL AND PRACTICE**					
[51]	Neurocognitive deficits	Neurocognitive enhancement therapy (NET) & working therapy (WT)	Cognitive flexibility, social inference, emotion recognition, abstract thought, verbal learning, memory	NET + WT greater improvements in executive functioning, working memory and affect recognition	Control group N = 65
[52]	Working memory deficits	CR and working therapy (WT)	Attention, memory and executive functioning	CRT+WT yield greater improvements and effects remain over time (6 months)	Control group N = 102
[53]	Cognitive deficits to improve work outcomes	Neurocognitive enhancement therapy (NET) + work therapy	Work productivity (hours and dollars earned)	Patients worked more hours, had more dollars earned and tended to have more competitive-wage employment	Control group N = 145
[54]	Attention, memory and executive functioning	Neurocognitive enhancement therapy (NET) + Work therapy Work therapy alone	Working memory, verbal and nonverbal memory, thought disorder, executive functioning	Significant improvements in working memory and executive functioning.Both groups had a significant effect on memory (verbal and visual)	Control group N = 145
[55]	Functional outcomes (follow up study using the same NET program so classified here instead of in Table 2)	Neurocognitive Enhancement Therapy (NET) + vocational program (VOC)	Work hours, employment rates	NET+VOC patients worked more hours during the 12 month follow-up period and they had higher rates of employment	Control group N = 72
[56]	Neurocognition, negative symptoms, self-esteem	Computer-assisted cognitive rehabilitation (CACR)	Attentional deficit, verbal and auditory memory, general level of cognitive functioning, negative symptoms, self-esteem	CACR improved verbal/conceptual learning and memory and executive functioning	Placebo group N = 34
[57]	Repetition and memory	Virtual reality training	Orientation, attention, calculations, constructions, memory, language, and reasoning	Improvement of overall cognition	Control group N = 27
[58]	Attention/concentration, working memory, logic, and executive functions	CR	Attention/vigilance, verbal/non-verbal working memory, verbal and visual learning and memory, speed of processing, reasoning, problem-solving, quality of life and social autonomy	Improvements in attention/vigilance, verbal memory, problem solving	Control group N = 77
[59]	Cognitive deficits	Pharmacotherapy and cognitive retraining (CR) together 1) drug+CR, 2) drug + control CR, 3) placebo + CR, 4) placebo+control CR	Verbal working memory, attention/vigilance Measures of tolerability and safety	CR- significant improvement in verbal working memory. Trend toward improvement in Attention/Vigilance	Control groups N = 104
[60]	Executive functioning (and metacognition)	Problem Solving and Cognitive Flexibility trainin (REPYFLEC)	Verbal and visual memory. cognitive flexibility, inhibition of impulsive responses, planning and organization, working memory and time-estimation capacity, attention, processing speed and cognitive flexibility social behavior and relationships, autonomy, employment-occupation and leisure, self-care, social behavior and autonomy	Significant improvements in executive function, negative symptoms and Positive change in life skills and psychosocial functioning. Skills maintained at follow-up especially in self-care, social behavior and employment-occupation.	Control group N = 62
[61]	Attentional deficit	Computer-Assisted cognitive rehabilitation or computer games	Various measures of attention such as trail making, letter-cancellation, Stroop, seach-a-word, etc.	Both groups improved in letter-cancellation task due to practice effect	Control group N = 10
[62]	Verbal and global cognition	Auditory training	Global cognition, speed of processing, verbal memory/learning, problem-solving, nonverbal memory, visual learning/memory, social cognition	Strong improvement in verbal and global cognition	Placebo group N = 55
[63]	Cognition in general	Targeted cognitive training (TCT)	Global cognition, speed of processing, verbal working and learning memory and cognitive control	TCT improvements in verbal learning/memory and cognitive control even 6 months after therapy	Control group N = 32
[64]	Cognitive deficits in memory	Computerized cognitive remediation training - digits sequenced recall and words sequenced recall (control: work therapy only)	Cognitive deficits, more specifically memory	Significantly greater improvements on the computerized memory task (digits sequenced recall) remained at the 6 month follow up	Control group N = 94
[65]	Memory, attention, cognitive flexibility	Vocational Program (VOC) and NET+VOC	Cognitive flexibility and executive functioning, working memory, visual and verbal memory, social cognition	VOC+NET greater improvement on all outcomes. No improvement in affect recognition after 1 year	Placebo group N = 72
[66]	Neural correlates of emotion identification	Training of Affect Recognition (TAR) and TAU	Emotion identification, emotion discrimination, digit symbol, digit span, symptoms, neural activation	TAR improved performance in emotion recognition and discrimination more than TAU and controls. Psychopathological status improvements for both TAR and TAU	Control group and healthy controls N = 30
[67]	Effects of age on cognitive functioning	CR and TAU	Working memory, cognitive flexibility and planning. Groups split on age	CR improved working memory only in younger group	Control group N = 134
[8]	attention, memory, language and problem-solving	CR and computer-skills training	Working memory, verbal episodic memory, speed of processing, visual episodic memory, reasoning and problem-solving	CR improved working memory but both groups showed improvement on other measures	Placebo group N = 42
[68]	Cognitive functioning in general	CR	Attention, psychomotor speed, verbal working memory, verbal learning and memory and executive functioning, information processing speed, academic achievement	Cognitive remediation improvements in overall cognitive functioning, psychomotor speed, and verbal learning	Control group N = 85
[69]	Cognitive functioning	Attention Process Training (APT)	Attention, memory and executive functioning Other: positive and negative symptoms	Neither group improved in symptoms and attention and memory measures. APT group had higher performance on executive function	Placebo group N = 24
[70]	Attention and information processing	Continuous Performance Test (CPT)	Attention and negative symptoms	CPT improved both measures	Control group N = 54
[71]	Memory	Memory remediation (MR), problem-solving remediation and TAU	Memory, verbal learning, problem-solving	MR improved memory but not verbal recall	Control group N = 54
[72]	Cognitive impairment	Brain Fitness Program (BFP)	Cognitive performance (CogStat) Functional capacity, auditory processing speed for verbal and non-verbal tasks	BFP training improved auditory processing speed but no effect on cognitive impairments	None N = 55
[73]	Divergent thinking	Rock-paper-scissors task, calculation tiles task	Idea, design and letter fluency, digit span, social functioning	Improvements in idea fluency, functioning, and interpersonal relations	Control group N = 17
[74]	Visual motion processing	Target discrimination	Perceptual motion and direction processing	Greater perceptual improvement in schizophrenia	Healthy controls N = 27
[75]	Cognitive and daily functioning deficits (but concentrating on the neurobiological mechanism that underline them)	CR and Social Skills Training	Functional and structural connectivity brain changes	Brain networks activation pattern significantly changed in patients exposed to the cognitive treatment in the sense of normalizing toward the patterns observed in healthy control subjects	Control groupN = 30
[76]	Dysfunctional organization of the auditory/verbal system	Targeted auditory/verbal discrimination Training (TAD) or CRT (CogPack)	Verbal learning and fluency, recall, working memory, clinical symptoms as exploratory measure	Improvement in verbal learning and memory for TAD but no effect on clinical symptoms	Control group N = 39
[77]	Brain oscillary activity, linked to dysfunctional information processing	Specific cognitive exercises (CE) fostering auditory/verbal discrimination or standard broad-range cognitive training (CP)	Verbal memory, global functioning, brain oscillary activity	CE improves brain oscillary activity and reduces information processing dysfunction	Control group and healthy controls N = 51
[78]	Verbal memory and learning, processing speed, working memory and attention	CR	Verbal memory, visual working memory, visuo-spatial memory, processing speed, psychomotor speed, working memory, verbal fluency, attention, visual-perceptual function	Patients in all groups improved in measures of information processing, verbal memory, and visuospatial memory	One placebo group and one control group N = 44
[79]	Cognitive deficits	CR (Cogpack)	Memory functions, attention, concentration, logical abilities, verbal reasoning	Cogpack improves cognitive functioning in persons at risk. Specifically at risk group improve in long-term memory functions, attention, and concentration. Patients with schizophrenia – no improvement.	Control group N =16 schizophrenia N = 10 at risk
[80]	Planning and problem-solving, processing speed, memory and attention	Plan-a-day And Training for basic cognition	Planning ability, problem-solving, global assessment, functional capacity, working memory, verbal memory, processing speed and inhibition	Both groups improved in measures of cognitive functioning and functional capacity. Plan-a-day improved planning	None N = 89
[81]	Verbal learning and processing speed	CR	Word fluency, memory and recall,	All outcomes improved in CR	Control group N = 42
[82]	Impairment in reality monitoring	CR	Reality monitoring Prefrontal cortex activity	Improvement in reality monitoring that correlated with increased medial prefrontal cortex activity (related to improvement in social functioning 6 months later)	Control group N = 31 (schizophrenia) N = 15 healthy controls
[83]	Visual and auditory learning	CR consisting of visual, auditory and cognitive control	Visual memory, visual-spatial memory, auditory verbal memory, verbal and letter learning	Visual training strongly predicts visual learning but not auditory learning	Placebo control N = 14
[84]	Perceptual, memory and motor functions	Sustained and repeated training with no instructions, increasingly demanding tasks	Visual word, visual dot localization, motor processing	After training, most participants performed as well or better than best controls on tasks	Control group and healthy controls N = 22

*Note.* CR = cognitive remediation. NEAR = Neuropsychological Educational Approach to Remediation. TAU = treatment-as-usual, NET = Neurocognitive Enhancement Therapy.

**Table 2:** Training to improve sociocognitive deficits

**DRILL AND STRATEGY**					
**Authors**	**Targeted deficits**	**Type of training**	**Measured variables**	**Results**	**Control and samples**
[85]	Social context appraisal	Social cognition enhancement training (SCET) and standard psychiatric rehab	Perceptual organization and sequencing in social contexts, emotion recognition	In SCET, some variables improved after 2 months, others after 6 months	Control group N = 34
[86]	Social cognition deficits	social cognition and interaction training (SCIT) and Control: coping skills groups	Emotion and social perception, theory of mind, attributional style, cognitive flexibility, and social relationships	Improved in all sociocogntive measures. Better self-reported social relationships	Control group N = 28
[87]	Emotion perception, attributional style, and theory of mind	SCIT and coping skills groups	Facial emotion identification and discrimination, social perception, theory of mind, attributional style and ambiguity, cognitive flexibility	Improvement in all aspects for participants in SCIT	Control group N = 18
[88]	Social cue recognition	Vigilance+memory training or vigilance alone	Social cue recognition	Better recognition of social cues in vigilance+memory	Control group N = 40
[89]	Emotional intelligence	Cognitive enhancement therapy (CET) and enriched supportive therapy (EST)	Emotional Intelligence	CET group improved in emotional intelligence	Control group N = 38
[90]	Learning and interpretation of social situations	Stimulus identification, interpretation of images and assignment of title	Sustained and selective attention, functional outcome, social perception	Improvement in all variables in therapy group, maintained at 6 months	Control group N = 18
[91]	Perception and interpretation of social situations	Integrated Psychological Therapy (IPT)	Social perception, attention, psychopathology and social functioning	IPT improved social perception. No differences in attention or symptoms between groups	Control group N = 20
[92]	Emotion perception	Emotion Management Training (EMT) or problem-solving	Emotion perception in self and others, social adjustment, coping strategies, psychopathology	EMT improved emotion perception, social adjustment and psychopathology. At 4 month follow up, gains maintained in social adjustment and psychopathology only	Control group N = 22
[93]	Social cognitive skills	Presentations, group practice and training exercises	Facial emotion identification, social perception, attributional style, theory of mind, speed of processing, attention/vigilance, working memory, verbal and visual learning, reasoning, problem-solving and social cognition	Improvement in facial affect perception only	Control group N = 31
[94]	Social cognitive deficits	Socio-cognitive skills training (SCST) Other conditions 1: Cognitive Remediation (CR) 2: standardm illness management skills training, 3: Hybrid treatment that combined elements of SCST and neurocognitive remediation	Emotional processing, social perception, attributional bias, and mentalizing	The SCST group demonstrated greater improvements over time than comparison groups in the social cognitive domain of emotional processing, including improvement in measures of facial affect perception and emotion management.	Control group N = 68
[95]	Theory of Mind (ToM)	Analyses and reasoning about social interaction scenes	ToM, symptoms, psychopathology, attribution	Slight improvement in ToM (not significant) in training group from first to second training session. No improvement in symptoms	Control group N = 14
[96]	Emotion perception	CR and computerized Emotion Perception intervention compared with CR only	Emotion recognition, emotion discrimination, personal and social performance (also neurocognition)	Combined CR with emotion perception remediation produced greater improvements in emotion recognition, emotion discrimination, social functioning, and neurocognition	Control group N = 59
[97]	Emotion recognition and ToM	Emotion and ToM Imitation Training and problem-solving	Psychopathology, symptoms, emotion recognition, ToM, neurocognition, flexibility, social functioning, attribution, neurophysiological activation	Training improved sociocognition (strongest was emotion recognition) and social functioning	Control group N = 32
[98]	Social cognition	State reasoning training for social cognitive impairment (SOCog-MSRT)	Theory of mind, Social understanding, Inference of complex mental states from the eyes Working memory, IQ	Improvement in ability to reason causally about false beliefs, to infer complex mental states from the eyes, and to intuitively understand social situations. However individuals with poorer working memory and lower premorbid IQ did not benefit	None N = 14
[99]	Social cognition	SCIT	Emotion perception, attributional style and theory of mind	Improved emotion perception, improved theory of mind, and a reduced tendency to attribute hostile intent to others	None N = 17
[100]	Emotion perception, ToM and social skills	SCIT and Treatment-As-Usual (TAU)	Emotion perception, theory of mind, attributional style, social skills in role-play	SCIT+TAU improved emotion perception but improvements on theory of mind inconsistent	Control group N = 31
[101]	Visual attention and facial emotion perception	CR and repeated exposure	Emotion recognition	Improvements in pre-post- means for CRT and maintained one month post-training	Control group N = 40
[102]	Emotion recognition and social perception	Social Cognitive Training Program and TAU	Emotion recognition, psychopathology, social functioning, social perception	Training improved social perception between group but no improvement in emotion recognition	Control group N = 14
[103]	Emotional communication, (Perception of facial emotional expression)	Computerized emotion training program	Identification of emotions, differentiation of facial emotions, working memory	Compared to baseline significantly better at identification of facial emotions. No changes in differentiation of facial emotions and working memory	None N = 20
[104]	Social cognition and quality of life	Family-social-cognition and social stimulation (F-SCIT)	Memory, visual-spatial scanning, divided attention, inhibition, emotion perception, theory of mind, empathy, reasoning, attributional style, insight, social functioning, quality of life	F-SCIT improved social withdrawal, interpersonal communications, prosocial activities, independence/competence, theory of mind, emotion perception	Control group N = 52
[105]	Social and emotion perception	CR	Emotion and general perception, attention, memory, executive functioning, visual processing, cognitive flexibility and interference	Improvement of emotion perception and executive functioning, other areas of neurocognition not affected	Placebo group N = 42
**DRILL AND PRACTICE**					
[106]	Deficits in facial affect recognition	Training of affect recognition (TAR) Controls groups: (TAU or CR)	Facial affect recognition, face recognition, and neurocognitive performance	Patients under TAR (but not CRT or TAU) significantly improved in facial affect recognition. Patients under CRT improved in verbal memory functions.	Control groups N = 77
[107]	Prosodic affect recognition, theory of mind	Training of Affect Recognition (TAR) and CR	Facial affect recognition, prosodic affect recognition, theory of mind, social competence in role-play	Larger pre- post- improvements on TAR for all variables	Control group N = 38

*Note*. SCIT = social cognition and interaction training. TAU = treatment-as-usual. CR = cognition remediation.

**Table 3:** Training to improve both neuro- and sociocognitive deficits

**DRILL AND STRATEGY**					
**Authors**	**Targeted deficits**	**Type of training**	**Measured variables**	**Results**	**Control and samples**
[27]	Social competence (interest, affect, fluency, clarity, focus) and neurocognition	Cognitive Remediation (CR) and Functional Adaptation skills training (FAST) Control: FAST or CR	Functional competence, information processing, verbal fluency, working memory, executive functioning, verbal memory	The early-course group had larger improvements in measures of processing speed and executive functions, adaptive competence and real-world work skills. Verbal memory, verbal fluency and social competence did not improve	None N = 39
[26]	Cognitive deficits and functional competence deficits	CR + skills training CR + Treatment-As-Usual (TAU) Skills training + TAU	Cognitive performance (reasoning, problem solving, processing speed, verbal memory, working memory)Social competence, functional competence, real-world functional behaviour	CR produced robust improvements in neurocognition, but not after functional skills training.Social competence improved with both type of training. Functional competence higher and more durable with combined treatment. Functional competence and real-world behavior was more likely when supplemental skills training and cognitive remediation were combined.	Control group N = 107
[108]	Neurocognition, social cognition and symptoms	Cognitive Enhancement Therapy (CET) or Enriched Supportive Therapy (EST)	Neurocognitive ability and processing speed, social cognition and cognitive style, social adjustment and symptomatology	CET improved social cognition, cognitive style, social adjustment and symptomatology during first year and neurocognition benefits were after 2 years	Control group N = 58
[109]	Sociocognition: social and emotional perception, attention, concentration, verbal memory	One program including 1) CR for neurocognition + 2) Social Skills Training for sociocognition and TAU	Verbal and non-verbal memory, attention, memory, executive functions, verbal fluency, self-care, underactivity, slowness in task execution, social withdrawal, participation in family life, functional outcome	Better efficacy in all measures for combined program compared to usual program	Placebo group N = 60
[110]	Organization, comparison and organization, orientation in space, relations, social skills, integrative thinking	CR on specific areas: organization, social skills, categorization	memory, thought process and self-concept, functional outcome	Experimental group showed improvements in cognitive abilities and daily functioning, no difference in self-concept	Placebo group N = 58
[111]	Sociocognition and neurocognition	Cognitive enhancement therapy (CET) or enriched supported therapy (EST)	Processing speed. neurocognition, cognitive style, social cognition, social adjustment and symptoms	12 months: improvement in neurocognition and processing speed 24 months: Same as 12 months and increase in cognitive style, social cognition and social adjustment	Control group N = 121
[112]	Neurocognitive and social-cognitive deficits	Cognitive enhancement therapy (CET) Enriched supportive therapy (EST)	Processing speed, Neurocognition, social cognition, cognitive style, social adjustment	Significant effect of CET on measures of processing speed, cognitive style, social cognition, and social adjustment. Only the neurocognitive composite is not significant at 36 months follow-up compared to the two years follow-up.	Control group N = 106
[113]	Symptoms, social adjustment, social cognition, cognitive style, neurocognition processing speed	CR and enriched supportive therapy (EST)	Symptoms, social adjustment, social cognition, cognitive style, neurocognition processing speed	Improvement in all domains for schizoaffective and schizophrenia patients. Except for schizophrenia, no improvement in processing speed	Control group N = 58
[114]	Neurocognition and sociocognition	Computerized neuroplasticity-based auditory training and Social cognition training (SCT)	Auditory perception, emotion identification, social perception, theory of mind tasks, all measures of the MATRICS	Gains in neurocognition Gains in emotion identification, social perception, and self-referential source memory.	None N = 19
[115]	Cognition (attention, memory), social perception, cognitive differentiation	CR + psychoeducational programme Psychoeducational programme	Symptoms, psychosocial functioning, attention, memory, executive functioning	Improvement in psychosocial functioning, reduced symptoms (except negative symptoms) and Improvements were observed for 8 of the 10 cognitive measures. Only verbal long term memory and executive functioning (cognitive flexibility) did not improve	Control group N = 25
[116]	Cognitive differentiation, attention, memory and social perception	CR	Symptoms, psychosocial functioning, attention, memory, executive functioning	Reduced in symptoms and psychosocial functioning, only verbal long term memory and executive functioning did not improve	Control group N = 25
[117]	Social cognition and problem solving, planning and memory	Cognitive-emotional rehabilitation (REC) and Problem Solving Training (PST)	Social and occupational functioning, working memory, psychomotor speed, verbal memory, executive functioning, verbal fluency, theory of mind	PST improved planning and memory, REC improved theory of mind and emotion recognition	None N = 24
[118]	Selective and Sustained attention, memory, conceptualization abilities, cognitive flexibility, social perception, verbal communication, social skills, and interpersonal problem solving	Cognitive remediation component of IPT	General attention, verbal memory, working memory, executive functions. Global social functioning, positive negative symptoms	Improvements verbal and working memory, improvements in negative and total symptom severity. Functional outcome mediated by improvement in cognitive domains	Control group N = 32
[119]	Selective and Sustained attention, memory, conceptualization abilities, cognitive flexibility, social perception, verbal communication, social skills, and interpersonal problem solving	Cognitive remediation component of IPT (IPT-cog) or computer-assisted cognitive remediation (CACR) Or rehabilitative interventions	Processing speed, working memory, memory in general, executive functioning, global social cognition	IPT and CACR improvements in all variables especially speed and processing and working memory and increase in functioning	Control group N = 90
**DRILL AND PRACTICE**					
[120]	Attention, executive functioning, memory quality of life, interpersonal relations, social abilities, autonomy	CR and Standard Rehabilitation Training (SRT)	Verbal + working memory, psychomotor speed and coordination, selective and sustained attention, semantic and letter fluency, cognitive flexibility, daily functioning, interpersonal relations	CR + SRT improvements on executive function, attention and daily functioning	Control group N = 86
[121]	Emotion recognition deficits in the neural mechanisms involved in emotion recognition	Auditory-based cognitive training (AT) (Brain Fitness), social cognition training or non-specific computer games (CG).	Recognition of negative and positive emotions Poscentral gyrus activity (neural region known to support facial emotion recognition)	Greater pre-to-post intervention increase in postcentral gyrus activity during emotion recognition Results indicate that combined cognition and social cognition training impacts neural mechanisms that support social cognition skills.	Placebo group N = 22

*Note.* CRT = cognitive remediation training, CBT = cognitive behavioral therapy, TAU = treatment-as-usual, MATRICS = Measurement and Treatment Research to Improve Cognition in Schizophrenia.

### Neurocognitive deficits

We identified a total of 62 studies pertaining to neurocognitive training. Of these, 58 included randomized controlled trials or placebo conditions, while four had no control. At first glance (see Table 1), it appears that for people with schizophrenia drill and practice training is used more frequently to train neurocognitive deficits (i.e., drill and practice = 35 studies, 33 with controls and two without; drill and strategy = 27 studies, 25 with controls and two without).

Examining the drill and strategy studies, a pattern rapidly emerges when the methods of training are considered. Twelve of 27 studies used group therapy in their training rather than individual computerized training with therapist assistance. However, there does not seem to be a link between the method of training (individual or group) and the outcome measures. Though it is not the goal of our review, it is important to note that all articles with drill and strategy approaches to training reported between-group improvements of the targeted deficits. Furthermore, eight of the 17 studies with follow up measures at either three, four or six months also reported sustained gains in cognition [25,32,37,41,45,47,48,50],[64].

Drill and practice studies most commonly used computerized tasks, done individually. However, there was more variety in the methods of training, for example, at least five studies used pencil-and-paper procedures [60,67,69,73,75]; though Lopez-Luengo utilized both pen-and-paper and audio] while five others used a combination of audio and visual tasks [62,63,77,78,83] to reduce the deficits. Furthermore, most studies using drill and practice methodologies (all except [61,69]) reported between-group improvements in cognition between the experimental and control groups, at least for some measures.

The studies we analyzed targeted a variety of neurocognitive deficits - memory, attention/vigilance, reasoning, verbal learning - yet overall, across studies, no single deficit stood out as being resistant to implicit training. Therefore, it would seem that most domains of neurocognition respond well to drill and practice training, even though only seven studies had follow ups at six months, six [52,55,60,63,64,82] confirming that the gains were maintained and one [65] showing that only the affect recognition benefits were not maintained at the 1-year follow up.

### Sociocognitive deficits

In contrast to studies focusing on neurocognition, those aiming to improve sociocognitive deficits used mostly drill and strategy approaches (i.e., drill and practice = two studies with control groups; drill and strategy = 21 studies, 18 with controls and three without). Importantly, all studies included a variety of visual aids such as vignettes, Powerpoint presentations or videos of social situations. Furthermore, visual presentations and explanations by the therapist about the goal of the training were often done in group settings. This method allows modelling by the therapist but also incorporates group exercises and practice as well as role-plays.

Interestingly, for sociocognition, whether the training paradigm was drill and strategy (e.g. [97]) or drill and 210 practice (e.g. [107]), there was a general concern to assess whether remediation of a specific type of deficit would generate generalizable results, not only to functional outcomes but also to broader domains of social cognition such as Theory of Mind.

### Studies that aimed to improve both neuro and sociocognition

It is more difficult to find a pattern in the types of training when the target deficits are broader and span across both neurocognitive (such as memory and attention) and sociocognitive domains (such as social perception and emotion recognition). However, most use drill and strategy paradigms that generally combine computer-assisted programs for neurocognition, and guided practice, modeling and role-play for sociocognition. There is also a mix of individualized and group approaches that seem, again, to follow the trend that neurocognition is trained individually while sociocognition is trained in groups, and this is true for both drill and practice as well as drill and strategy.

## Discussion

The purpose of this article was to review the type of training – whether drill and practice or drill and strategy – most often offered in clinical studies to people with schizophrenia to help overcome neurocognitive or sociocognitive deficits. We included articles with varying scientific value for both neurocognitive and sociocognitive training; nine of the 99 articles we reviewed had no control condition. However, since we are not presenting a thorough analysis of the efficacy or effectiveness of these training methods (see 14 for details, [22,23]), we opted to include them for descriptive purposes. Although we found a variety of training modalities offered, some more behavioral, some using computer training, real-life situations, indirect training, etc., we were able to determine if a training paradigm was drill and practice or drill and strategy in nature, and which of these methods was used more frequently to improve neurocognitive or sociocognitive deficits. We also planned to describe the patterns and modalities used to train the targeted deficits (i.e., neuro- or sociocognitive).

In our literature search, we found that drill and practice training programs were used more frequently for improving neurocognitive deficits. Of the 62 studies we reviewed, 35 used procedures that mostly involved errorless learning, a type of training where the degree of difficulty of the task increases with the performance of the participant and where no conscious effort is necessary to improve. Studies using drill and strategy (n = 27) seemed particularly interested in the impact of the training on other variables outside of neurocognition, such as symptoms and quality of life. This was not the case for the drill and practice approaches. Another difference was that studies using drill and strategy training almost always measured executive functioning (n = 15), whereas studies using drill and practice training did not. However, we could not determine whether one specific domain of neurocognition was more easily retrained than another with drill and practice vs. drill and strategy procedures. Furthermore, most studies were of short duration and only a few had follow up measures (e.g., drill and strategy n = 8 [25,32,37,41,45,47-49]; drill and practice n = 7 [52,55,60,63-65,82]. This could be improved upon in future studies, since it is difficult under these circumstances to decide whether the observed effects are maintained over time or not.

When attempting to put the findings on neurocognitive deficits into context, we wondered why drill and practice training would be used more often to retrain neurocognitive deficits. The answer may lie in the way these functions interact in our cognitive processes. Some domains, like attention and speed of information processing, seem more implicit by nature – the bottom-up approach. We could posit that these functions are not used consciously and a person would not need to inherently know “how” to use the functions; instead they would simply perform the task repetitively and unconsciously. However, this might imply that drill and practice procedures would only improve neurocognitive deficits, which might not be the case, as judged by the results reported in recent meta-analyses [14,22]. Furthermore, since implicit learning has been reported as being generally intact in schizophrenia [84], some, like Fisher and colleagues [62], suggest that high levels of repetition (e.g., more than 1,000 rehearsals) and a high percentage of reward schedule (e.g.: 85%), will allow for neurological improvements. Yet, studies using drill and strategy procedures in their training methods also seem to generate consistent positive outcomes – the top-down approach. Of note, Wykes and colleagues [14] suggested that drill and strategy training include elements that are explicitly learned (through modeling, explanation or role-play – the “strategy”) but also elements inevitably linked with repetition (the “drill”) and considered implicit learning, which might explain why they are effective.

Tentatively, we suggest that since drill and strategy learning is thought to allow better integration of the rules and, thus, greater association between the various training elements [122], changes in cognition tend to occur over time. Blairy and colleagues [25], who also reported long-lasting improvements on memory and executive functions after explicit training, hypothesized that participants learned to bind different aspects of the experiment together and that it allowed for better consolidation in memory. Thus, at this time, we cannot draw a conclusion about whether certain domains of neurocognition respond better to one type of training over another. Further studies must be conducted, preferably comparing different forms of training with each other and adding follow up measures to assess whether the benefits of training remain stable through time.

Social cognition is considered by many researchers to have a strong relationship with positive functional outcomes [123,124]. Concurrently, the meta-analysis by McGurk [12] reported that programs using strategy coaching (drill and strategy training) for sociocognitive deficits had strong effects on functional outcomes as well as on the targeted social cognition skills. Consistent with this, we found that drill and strategy training was more frequently used for sociocognitive retraining. It seems intuitive that learning and integrating a social skill requires that it be practiced in a social setting, which was consistent with our findings when analyzing the studies. Most used group settings, where participants received their training then performed and practiced the learned techniques with a therapist to correct the behavior and give feedback. Moreover, it was also reported that integrating rehearsals into the training yields greater functional outcome improvements [23]. Indeed, sociocognitive studies tend to measure social functioning or social adjustment following training more often than studies aiming to improve upon neurocognitive deficits. Yet, a growing field around implicit learning in social cognitive psychology [125] suggests that drill and practice or other forms of more implicit training might be useful for sociocognition as well.

The collection of studies of Bell and colleagues on work and social outcomes using drill and practice [53,55] hint at the importance of generalizing the benefits of training to real-life situations, such as the ability to find and maintain work or to increase work productivity in the form of hours and money earned. However, both of these studies integrated the drill and strategy approach with a program of supported employment, creating a hybrid retraining program which has been efficient in the past [14]. Indeed, while improving cognitive deficits is commendable, functional outcomes are issues that should not be dismissed when considering the difficulties faced by individuals suffering from schizophrenia when trying to reintegrate the work force or create a social network.

We have also discovered that training programs usually target cognitive improvements “at large”, rather than specifically focusing on the individual deficits highlighted by the person’s profile, most likely to allow more people to receive the training without the need for specific neuropsychological or sociocognitive evaluations. We suggest choosing one type of training over another depending on the overall goal one is trying to achieve: drill and practice for precise deficits and drill and strategy to obtain general gains. More studies are needed to determine if drill and practice could be useful for sociocognition as well.

Furthermore, specific training methodologies seem to benefit specific domains of social cognition. For example, though it appears that Social Cognition and Interaction Training (even when including the family in the training sessions) improves Theory of Mind (ToM), group practices and Powerpoint presentations detailing the concepts of ToM did not improve ToM but did improve emotion recognition. We suggest that ToM is a more complex construct of sociocognition and requires more precise and detailed training than emotion recognition. Horan and colleagues [93] suggest that even defining the different concepts contained within ToM, such as appreciation of humour, is difficult and the training for it is more challenging. Furthermore, a recent meta-analysis of social cognition training in schizophrenia [21] also reported inconsistent effect sizes when ToM is targeted, suggesting that the key elements needed in the training for ToM must be better identified.

When the objectives of the training are broader, meaning that they aim to improve both neurocognitive and sociocognitive deficits through drill and strategy, the variables measured are also more varied and often include certain measures of functional or occupational outcome. Furthermore, these studies often tend to combine training with other types of intervention such as cognitive-behavior therapy, supportive therapy or occupational therapy.

Overall, our review summarizes the current state of research into cognitive training in schizophrenia. In neurocognition, drill and practice training is used more frequently and with a variety of different procedures such as auditory training [62] or target discrimination [74]. Tailoring the training to specifically address precise deficits might be one of the key benefits of drill and practice training. However, from the studies we evaluated, drill and strategy training was more easily generalized to all neurocognitive deficits. Indeed, a recent meta-analysis on the benefits of cognitive remediation in schizophrenia noted that this modality of training produces stable benefits on global cognition [14]. We suggest choosing one type of training over another depending on the overall goal one is trying to achieve: drill and practice for precise deficits and drill and strategy to obtain general gains in neurocognition.

### Limitations

There are a few limitations to our review. First, to reflect current trends, we included only studies published between 1995 and 2013, although interest in cognition remediation started as early as the end of the 1970’s [126]. Second, the fact that drill and practice or drill and strategy training can involve multiple strategies and training techniques (e.g., times eye tracking, computer programs, paper-pencil tasks, errorless learning, group learning, and various modalities of feedback) prevented us from describing them in detail and some of these specific strategies might explain differences in outcomes. Our goal was to describe what was being offered, not to promote one approach in particular. We also did not include studies described as “metacognitive”, a term that involves cognitive biases, at times social and/or neurocognitive, that are linked to the symptoms of psychosis [127] – for example, focusing on the cognitive bias of jumping to conclusions as linked to delusions. It is important to note that these types of training are not the only modalities offered to help overcome neurocognitive or sociocognitive deficits. Occupational therapy [128], social skills training [129], as well as certain forms of metacognitive psychotherapies [130] have also been documented.

## Conclusion

Future research is warranted to compare both drill and strategy and drill and practice programs with one another under control and experimental conditions, as well as to highlight the benefits and limitations of each. This would help to identify which type of deficit would benefit more from which training or to isolate particular participant profiles that respond best to a specific training strategy. Moreover, we suggest that more focus be brought to targeting participants’ specific deficits to tailor the training to those needs. This would increase the potential impact and generalization to “real-life” situations, both in the context of neuro and sociocognitive retraining. Finally, we propose investigating the benefits of both neurocognitive and sociocognitive training in the context of comorbidity. It is well know that schizophrenia is often comorbid with social anxiety (in 30% of cases; [131]) and substance abuse (in 50% of cases; [132]), to name a few. It is conceivable that the interplay of those disorders could be a substantial challenge for training. Nevertheless, very few studies have examined the impact of these presentations and doing so would be of paramount importance as it could increase the ecological validity and generalizability of the results.

### Endnote

^a^*stands for truncation.

## Competing interests

The authors declare that they have no competing interests.

## Authors’ contributions

KP conducted the literature search, selected and classified the appropriate articles, created the tables and wrote the manuscript.ALW read and classified the review articles to double-check KP’s previous work. She was also the first reader of some articles, in which case KP double-checked the classification. CC reviewed the comments from the reviewers and suggested improvements for the manuscript while answering the reviewers concerns. She also read and reviewed some articles that were missing from the first version of the manuscript. TL and SP are KP’s thesis director and co-director, respectively. They provided proof-reading, editing suggestions and feedback on the writing process. All authors read and approved the final manuscript.

## Pre-publication history

The pre-publication history for this paper can be accessed here:

http://www.biomedcentral.com/1471-244X/14/139/prepub
